# The MFN1 and MFN2 mitofusins promote clustering between mitochondria and peroxisomes

**DOI:** 10.1038/s42003-022-03377-x

**Published:** 2022-05-06

**Authors:** Yinbo Huo, Weiping Sun, Tiezhu Shi, Song Gao, Min Zhuang

**Affiliations:** 1 School of Life Science and Technology, ShanghaiTech University, Shanghai, 201210 China; 2 Shanghai Institute of Biochemistry and Cell Biology, Center for Excellence in Molecular Cell Science, Chinese Academy of Sciences, Shanghai, 200031 China; 3 University of Chinese Academy of Sciences, Beijing, 100049 China; 4 Hangzhou Institute for Advanced Study, University of Chinese Academy of Sciences, Hangzhou, 310007 China; 5 Department of Urology, Shanghai General Hospital, Shanghai Jiaotong University, Shanghai, 200080 China; 6 State Key Laboratory of Oncology in South China, Collaborative Innovation Center for Cancer Medicine, Sun Yat-sen University Cancer Center, Guangzhou, 510060 China; 7 Guangzhou Regenerative Medicine and Health Guangdong Laboratory, Guangzhou, 510530 China

**Keywords:** Peroxisomes, Mitochondria

## Abstract

Mitochondria and peroxisomes are two types of functionally close-related organelles, and both play essential roles in lipid and ROS metabolism. However, how they physically interact with each other is not well understood. In this study, we apply the proximity labeling method with peroxisomal proteins and report that mitochondrial protein mitofusins (MFNs) are in proximity to peroxisomes. Overexpression of MFNs induces not only the mitochondria clustering but also the co-clustering of peroxisomes. We also report the enrichment of MFNs at the mitochondria-peroxisome interface. Induced mitofusin expression gives rise to more mitochondria-peroxisome contacting sites. Furthermore, the tethering of peroxisomes to mitochondria can be inhibited by the expression of a truncated MFN2, which lacks the transmembrane region. Collectively, our study suggests MFNs as regulators for mitochondria-peroxisome contacts. Our findings are essential for future studies of inter-organelle metabolism regulation and signaling, and may help understand the pathogenesis of mitofusin dysfunction-related disease.

## Abstract (web summary)

The MFN1 and MFN2 mitofusin proteins localize to the outer mitochondrial membrane, where they may help functionally tether mitochondria to peroxisomes.

## Introduction

Peroxisomes are single-membrane organelles that function as a compartment for lipid oxidation and synthesis. It also plays a role in ROS (reactive oxygen species) metabolism by generating and removing ROS. Peroxisomes are structurally and functionally related to mitochondria in lipid and ROS metabolism and share similar fission machinery^1^. Proteins required for mitochondria fission, such as MFF, FIS1, and DNM1L, are also involved in peroxisome fission. So far, it is not known if any mitochondria fusion-related proteins are involved in peroxisome function.

Peroxisomes and mitochondria form organelle contacts in different ways^2,3^. Dual organelle targeting protein ECI2 contains N terminal mitochondria targeting sequence and C terminal peroxisome targeting sequence, therefore tethers mitochondria to peroxisomes^4^. In yeast, Fzo1 contributes to mitochondrion-peroxisome contacts, possibly through homo-dimerization^5^. It was also reported that *de novo* biogenesis of peroxisomes is mediated by the fusion of mitochondria-derived vesicles (MDVs) and ER-derived pre-peroxisomes^6^. More recently, a proteomic analysis of MDVs reveals the presence of both mitochondrial and peroxisomal proteins^7^.

Organelle contacting is ubiquitous and organelle clustering has been observed in various stressed conditions^8,9^. Mitochondria are clustered in the perinuclear region under the hypoxia condition in a microtubule and dynein-dependent manner^10^. MFN2 overexpression causes perinuclear clustering of fragmented mitochondria^11^. The presence of GTPase truncated MFN1 also causes a similar effect of mitochondria clustering^12^. Similarly, peroxisomes show homogenous cytosol distribution under normal conditions, while perinuclear clustering of peroxisomes has also been observed in PEX14 deficient cells^13^. Co-clustering of different organelles near the nucleus, including Golgi and mitochondria are also observed during apoptosis^14^. However, the correlation of different organelle clustering is not described. It is not known if any common regulators exist for different types of organelle clustering or co-clustering.

PEX2, PEX10, and PEX12 are three transmembrane ubiquitin ligases located on the peroxisome membrane^15,16^. All are required for peroxisome matrix protein importing. To further profile peroxisome surface protein, we applied a proximity tagging system PUP-IT to PEX ubiquitin ligase to identify their proximal proteins. Surprisingly, we identified many mitochondrial proteins in the proximity of peroxisomes. We further validated the interaction between PEX and mitofusins (MFNs). To further investigate the biological impact of MFNs on peroxisomes, we found overexpressed MFNs induced specific mitochondria-peroxisome clustering, and endogenous MFNs are enriched at the mitochondria-peroxisome contacting sites. Furthermore, leflunomide-induced upregulation of MFNs also triggers enhanced mitochondria-peroxisome contacting. Finally, we propose the role of mitofusins in regulating mitochondria-peroxisome clustering.

## Results

### The design of the proximity tagging system for PEX2/10/12

**Fig. 1:**
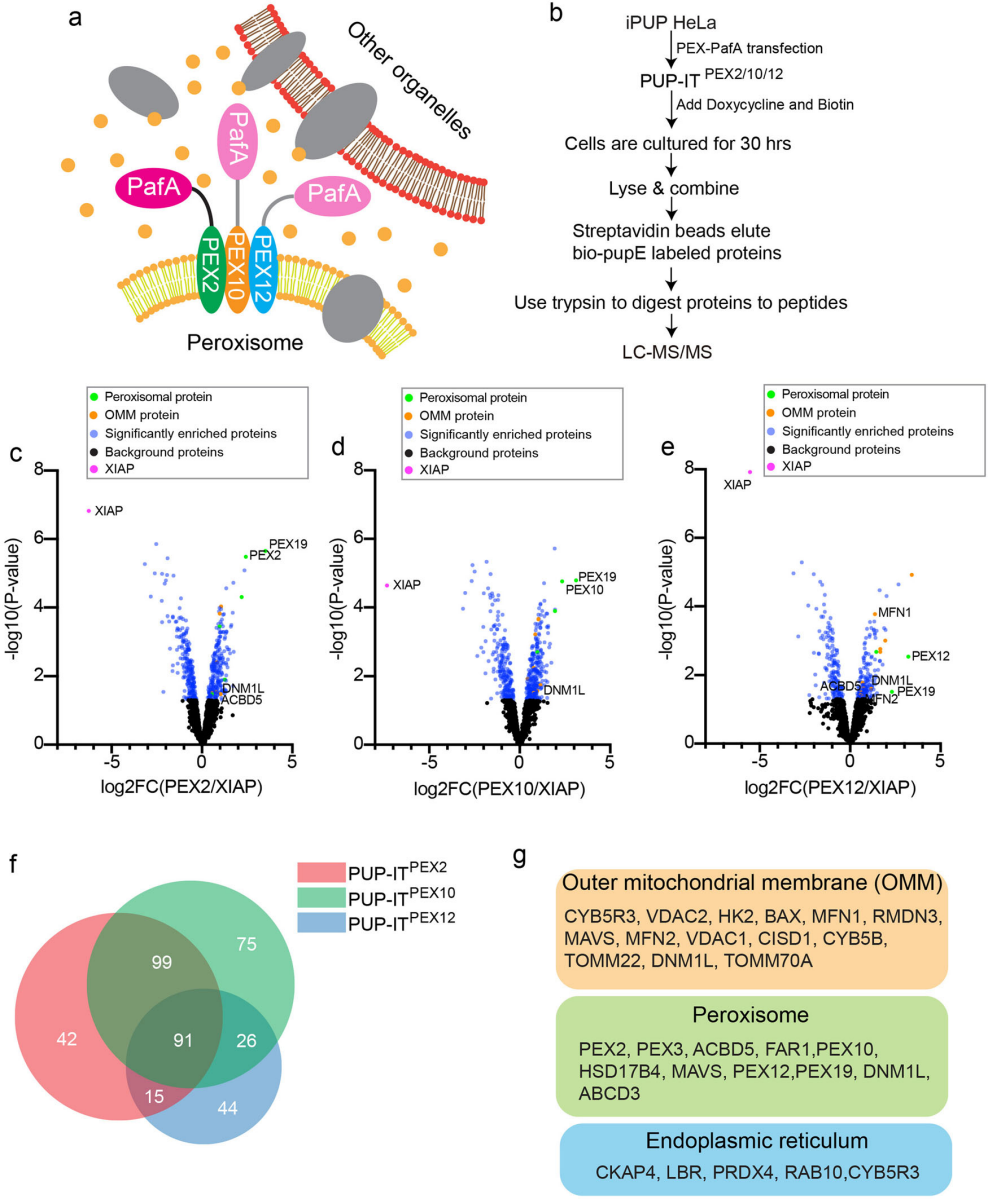
Identification of PEX2/10/12 interacting proteins by PUP-IT. **a** Schematic view of the PUP-IT design to identify PEX2/10/12 proximity proteins. PafA was fused to one of the PEX proteins, co-expressed with bio-PupE (yellow dots), and mediates bio-PupE modifications on proximal proteins. **b** The experimental workflow. The iPUP HeLa cells were transfected with PEX2-PafA, PEX10-PafA, and PEX12-PafA, respectively, to generate PUP-IT ^PEX2^, PUP-IT^PEX10^, and PUP-IT^PEX12^ HeLa cells. The proximity labeling was induced in 2 × 10^7^ cells by the addition of doxycycline (2 μg/ml) and biotin (4 μM) for 30 h. Cells were lysed, and bio-PupE modified proteins were enriched and identified by mass spectrometry. **c**–**e** Volcano plots of PUP-IT^PEX2^, PUP-IT^PEX10^, and PUP-IT^PEX12^-interacting proteins. The logarithmic ratios of protein label-free quantification intensity (PEX/XIAP) were plotted against negative logarithmic P values in the limma package. Limma-based t-test was used for significant difference testing of data. The candidate proteins were determined by a moderated t-test (*P*-value < 0.05) and fold change (fold change > 1.3). The blue dots represent significantly enriched proteins (false discovery rate ≤ 0.05; *n*  =  3 independent experiments). The right arm comprises proteins that are proximal to peroxisomes, the left arm proteins proximal to XIAP. Yellow dots represent outer mitochondrial membrane (OMM) proteins. Green dots represent peroxisomal proteins. The magenta dot represents XIAP. **f** Analysis of significantly enriched proteins in three datasets (PUP-IT^PEX2^, PUP-IT^PEX10^, and PUP-IT^PEX12^) through Venn diagram. The data were analyzed with BioVenn^41^. **g** Subsets of proteins identified with proximity labeling include OMM proteins, peroxisomal proteins, and ER proteins. Other proteins are not shown but included in the Supplementary Data 1. Protein localization was assigned based on Uniprot and MitoCarta 3.0^42^.

To identify potential peroxisomal membrane proteins in proximity to the PEX ubiquitin ligases, we applied the proximity tagging system PUP-IT to identify PEX2/10/12 interacting proteins. The PUP-IT system includes a protein ligase PafA and its substrate PupE, where PafA activates PupE and tags PupE to nearby proteins^17^. First, we generated a HeLa cell line containing BCCP domain-fused PupE under the TET-ON promoter (Supplementary Fig. 1a). BCCP (Biotin Carboxyl Carrier Protein) domain contains a lysine modified by biotin in mammalian cells. Therefore, we refer to BCCP-PupE as bio-PupE in the following text. In the presence of the TET-ON inducer doxycycline (DOX), the isolated single-cell clones all expressed bio-PupE (Supplementary Fig. 1b). We picked clone #5 for all the following proximity labeling experiments and named this cell line iPUP HeLa. We transfected iPUP HeLa cells with different PUP-IT designs. More specifically, we genetically fused the proximity ligase PafA to the C terminus of PEX2, PEX10, and PEX12 to generate PUP-IT^PEX2^, PUP-IT^PEX10^, and PUP-IT^PEX12^, respectively (Fig. 1a). A non-related ubiquitin ligase XIAP was also cloned into PafA fusion to be used as the control. PafA is active in all fusion forms, which mediates bio-PupE modification of various proteins showing different bands on western blots (Supplementary Fig. 1c). The fusion of PafA to PEX2, PEX10, or PEX12 does not affect the peroxisomal localization of these PEX ubiquitin ligases (Supplementary Fig. 1d).

We scaled up the proximity labeling experiment for affinity pulldown and mass spectrometry identification, using a standardized procedure demonstrated in Fig. 1b. For each PUP-IT labeling, we performed three biological repeats as described before^18^.

### Identification of PEX2/10/12 proximal proteins

Overall, we identified 392 proteins by mass spectrometry with proximity labeling, considering PEX2, 10, 12 as an intact complex. Using label-free quantification (LFQ) intensity, we compared the fold change of the LFQ intensity between experimental and control samples and ranked proteins enriched in each sample. By comparing with XIAP, we identified 247 proteins as potential PEX2 interacting proteins, 291 proteins as potential PEX10 interacting proteins, and 176 proteins as potential PEX12 binders (Fig. 1c–e and Supplementary Data 1). Notably, there are 91 proteins identified in all PEX-related samples, consistent with the common biological role of PEX2,10,12 in peroxisome protein translocation (Fig. 1f).

A few known PEX2, 10, 12 interacting proteins, including PEX19 and PEX3, were identified with this proximity tagging strategy (Fig. 1g). PEX2/10/12 are peroxisomal membrane proteins that are synthesized in the cytosol and recognized by PEX19. PEX19 binds to newly synthesized peroxisomal membrane proteins to dock on PEX3 on peroxisome and further assist the translocation of different membrane proteins^19,20^. PEX2, 10, 12 contain the PEX19 recognition sequence, therefore associate with PEX19 and PEX3. The PUP-IT system mediates self-labeling of the fused protein. PEX2, 10, 12 are identified by mass spectrometry as expected. A few other peroxisome proteins are also identified, including the peroxisomal membrane proteins ABCD3, ACBD5, and FAR1 (Fig. 1g). Considering those proteins are the most highly abundant peroxisomal membrane proteins, these results may reflect the nature of proximity labeling, where the proteins enriched on peroxisomes are labeled since peroxisomes are relatively small organelles.

Surprisingly, we also identified many proteins as potential PEX interacting proteins, including CYB5R1, MFN1, MFN2, TOMM70A, and TOMM22 et al. (Fig. 1g), indicating the proximity between PEX2/10/12 and some mitochondrial proteins. To exclude the possibility that over-expressed PUP-ITs are mislocated on mitochondria, we examined the cellular localization of PEX-PafA. All PUP-ITs show the overlapping localization with peroxisomes indicated by ABCD3 staining, but no apparent colocalization with mitochondrial marker COX4 (Supplementary Fig. 1e).

### Biochemical validation of PEX/MFN interactions

**Fig. 2:**
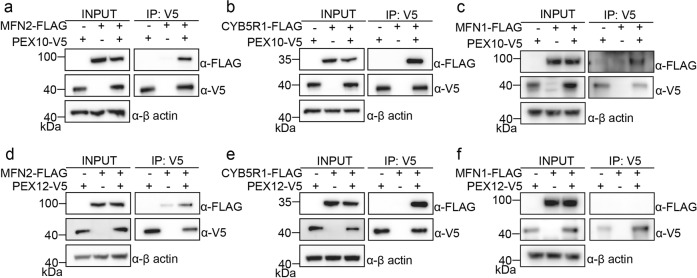
PEX10 and PEX12 interact with MFN proteins. **a** Co-immunoprecipitation (IP) of PEX10 and MFN2. V5 tagged PEX10 was co-transfected with FLAG-tagged MFN2 in HEK293T cells. Cells were collected 36 h after transfection for IP of PEX10-V5 and immunoblotting (IB) with indicated antibodies. Cell lysates and immunoprecipitated proteins were loaded with same amount on multiple gels for different blots. Similar co-immunoprecipitation experiments were performed for PEX10 and CYB5R1 (**b**), PEX10 and MFN1 (**c**), PEX12 and MFN2 (**d**), PEX12 and CYB5R1 (**e**), and PEX12 and MFN1 (**f**).

Among PUP-IT^PEX2/10/12^ proximity labeled proteins, we selected seven candidates for further validation, including CYB5R1, mitochondrial OCIAD1, MFN2, peroxisomal PEX19, ACBD5, and dual-organelle locating DNM1L and FIS1. In HEK293T cells, V5-tagged PEX10 was co-expressed with FLAG-tagged interacting candidates, immunoprecipitation of PEX10 was performed with anti-V5 antibody, the co-immunoprecipitated proteins were examined with anti-FLAG antibody. CYB5R1 and MFN2 showed a strong association with PEX10 (Supplementary Fig. 2a). Individual validation of MFN2 and CYB5R1 as PEX10 interacting proteins were also performed with more controls (Fig. 2a, b). Since MFN1 and MFN2 have significant functional overlaps, we also examined the interaction between MFN1 and PEX10 (Fig. 2c). PEX10 also associates with MFN1. Similar experiments were performed with PEX12. PEX12 associates with MFN2 and CYB5R1 (Fig. 2d, e). However, MFN1 does not associate with PEX12 in the traditional co-immunoprecipitation experiment (Fig. 2f). Since MFN1 peptides were detected by mass spectrometry for the PUP-IT labeling (Supplementary data 1) and PUP-IT is considered more sensitive for weak and transient protein–protein interactions, it is possible that MFN1 associates with PEX12 but under the detection limit of co-immunoprecipitation.

### The functional relation between MFNs and peroxisomes

Since MFN1 and MFN2 are both known as mitochondrial proteins, playing critical roles in mitochondria fusion. Deletion of mitofusins causes the defect of mitochondria fusion, while overexpressed mitofusins cause mitochondria fusion^21^. Giving we have identified MFNs as PEX10/12 interacting proteins, it is possible that MFNs regulate peroxisome morphology or function. Therefore, we examined the peroxisome shape with MFN overexpression.

**Fig. 3:**
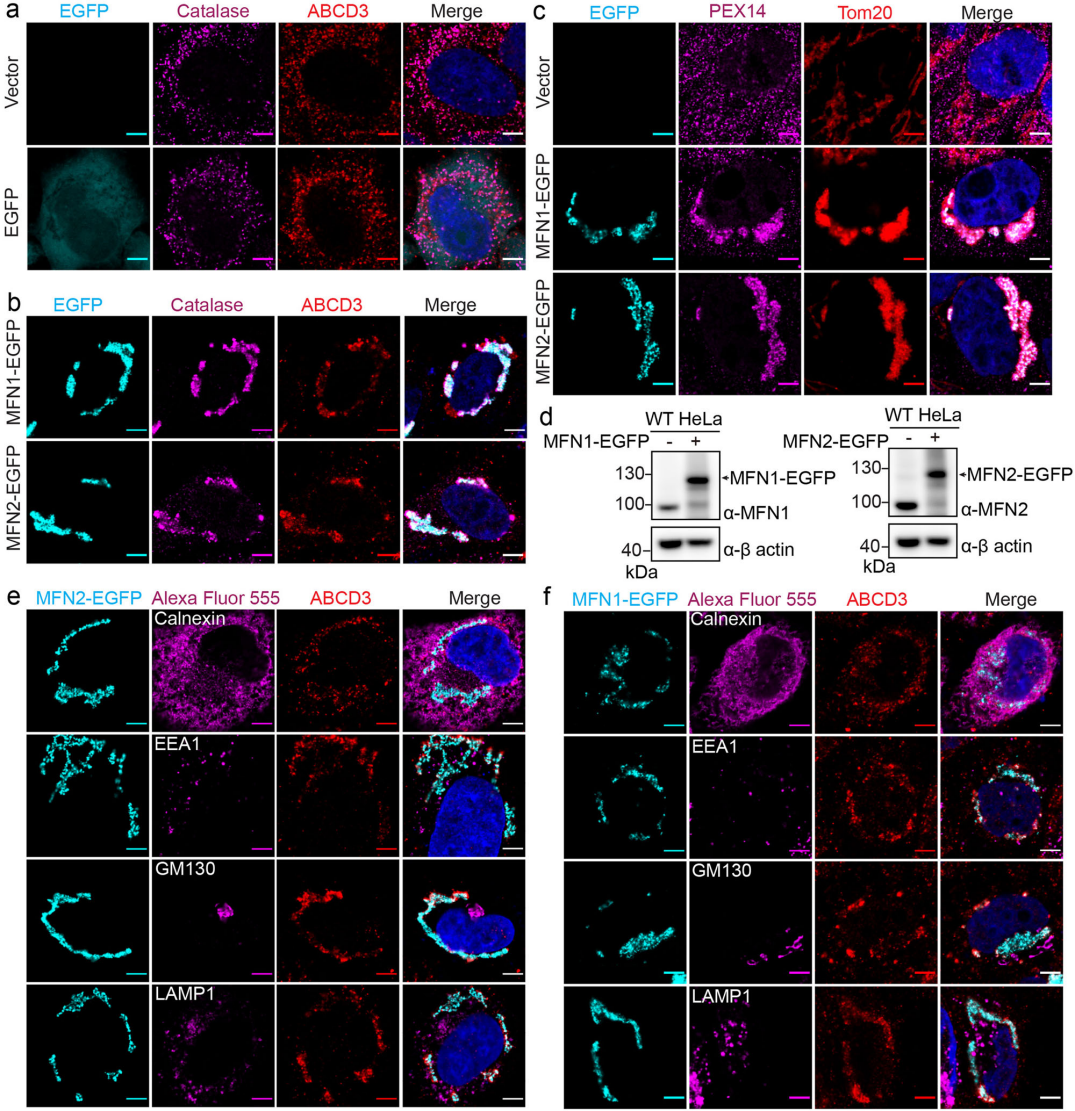
Exogenous expression of MFN induces peroxisome/mitochondrion clustering. Immunofluorescence images of overexpressed MFN-EGFP. HeLa cells were transfected with free EGFP (**a**) or EGFP fused MFN (**b**) plasmids for 36 h. Peroxisomal matrix protein catalase (Alexa Fluor 555) and peroxisomal membrane protein ABCD3 (Alexa Fluor 647) were immune-stained. Scale bars, 5 μm. **c** Peroxisomal membrane protein PEX14 (Alexa Fluor 555) and outer mitochondrial membrane protein Tom20 (Alexa Fluor 647) were immunostained with or without exogenously expressed MFNs. Scale bars, 5 μm. **d** Immuno-blots of overexpressed MFN1-EGFP and MFN2-EGFP. 1.5 µg plasmids were transfected into HeLa cells in one well in a six-well cell culture plate for 36 h and immunoblotted with indicated antibodies. **e** Immunofluorescence images of overexpressed MFN2-EGFP and other organelle markers. HeLa cells were transfected with MFN2-EGFP and immunostained for peroxisomal membrane protein ABCD3 (Alexa Fluor 647) and other organelle markers (Alexa Fluor 555): calnexin (endoplasmic reticulum), EEA1 (early endosome), GM130 (Golgi), and LAMP1 (lysosome). Scale bars, 5 μm. **f** Immunofluorescence images of overexpressed MFN1-EGFP and other organelle markers stained the same as in (**c**).

To observe peroxisome morphological change, we stained the peroxisomes with a peroxisomal membrane protein ABCD3 and a peroxisome matrix protein catalase. In wild-type HeLa cells or cells transfected with EGFP, the ABCD3 and catalase signals overlap well, showing dispersed dots representing individual peroxisomes (Fig. 3a). However, when MFN1-EGFP or MFN2-EGFP was overexpressed, ABCD3 and catalase signals still overlap well but show a clustered pattern accumulating around mitochondria, which are indicated by MFN-EGFP (Fig. 3b). To further confirm the clustering of mitochondria and peroxisomes, we used an alternative set of organelle markers, including Tom20 for mitochondria and PEX14 for peroxisomes. In the presence of exogenously expressed MFNs, immuno-fluorescent staining of Tom20 and PEX14 suggests the co-clustering of these two organelles (Fig. 3c). These results suggest the two types of organelles, mitochondria, and peroxisomes, are clustered when MFNs were overexpressed.

We wonder if the clustering is unique to mitochondria and peroxisomes. Thus, several other organelle markers, including calnexin for ER, EEA1 for early endosome, GM130 for Golgi, and LAMP1 for lysosomes, were used to determine the clustering status of those organelles. Unlike peroxisomes, none of these organelles clustered with mitochondria in the presence of MFN1-EGFP or MFN2-EGFP, indicating the unique role of MFNs to induce peroxisome-mitochondrion (PerMit) clustering (Fig. 3d, e).

**Fig. 4:**
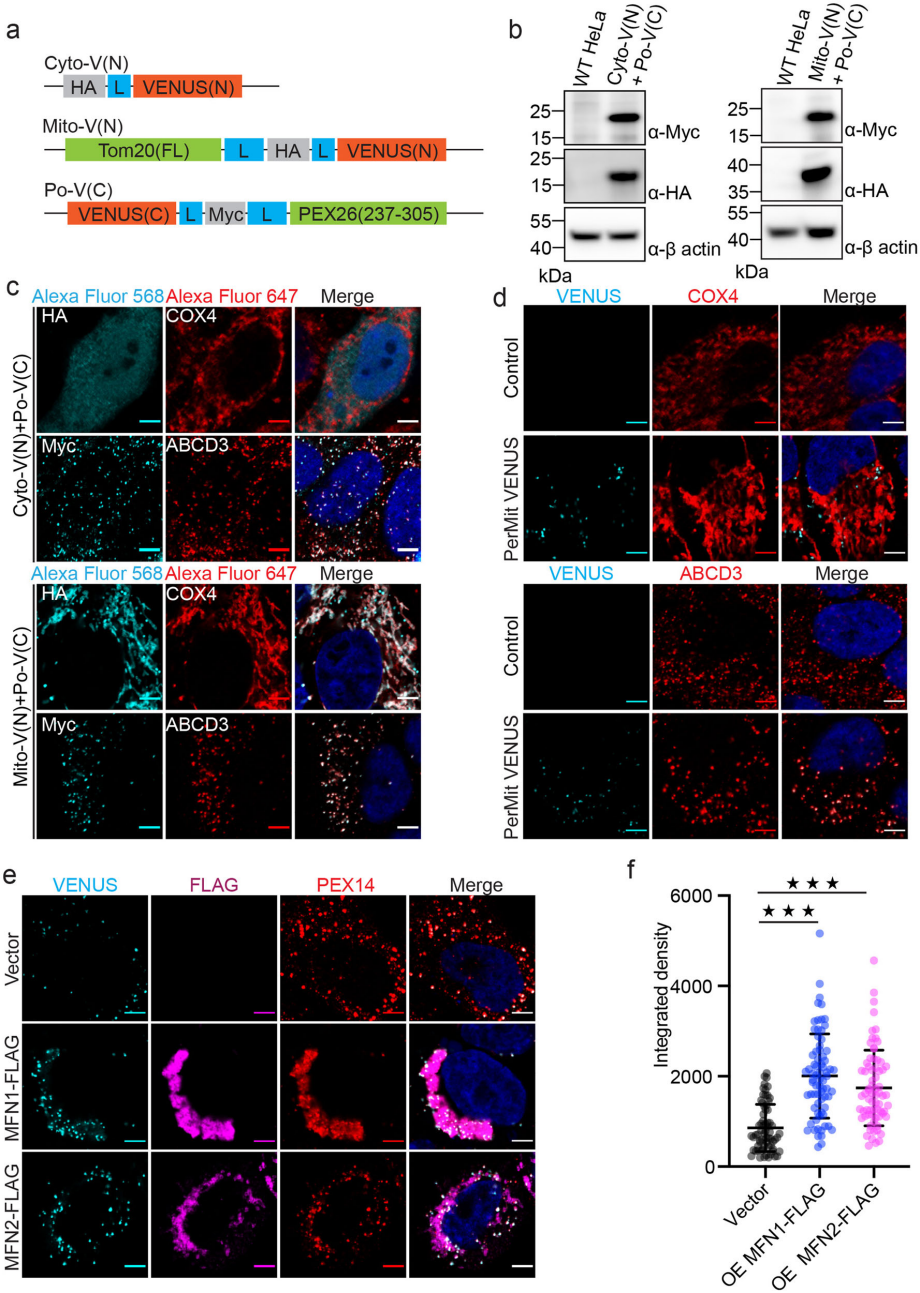
Exogenous expression of MFNs enhance the PerMit Venus reporter signal. **a** Schematic for the constructs of Cyto-V(N), Mito-V(N), and Po-V(C). Cyto-V(N), HA tag fused to the N terminus of Venus with a linker composed of 4 × GGSG (indicated with blue box); Mito-V(N), Tom20 fused to the N terminus Venus with a HA tag and two linkers as indicated; Po-V(C), Myc tag and PEX26(residues 237-305) fused to the C terminus of Venus (residues 155-238). **b** Immuno-blots for Cyto-V(N), Mito-V(N), and Po-V(C). **c** Immunofluorescence images of Cyto-V(N), Mito-V(N), and Po-V(C) co-stained with mitochondrial COX4 and peroxisomal ABCD3 in HeLa cells. Scale bars, 5 μm. **d** Immunofluorescence images of Venus co-stained with mitochondrial COX4 and peroxisomal ABCD3 in PerMit Venus and control cells. Scale bars, 5 μm. **e** Immunofluorescence images of PerMit Venus cells with exogenously expressed MFNs. PerMit Venus cells were transfected with empty vector, MFN1-FLAG, or MFN2-FLAG plasmids for 36 h. FLAG (Alexa Fluor 568) and peroxisomal membrane protein PEX14 (Alexa Fluor 647) were immunostained. Scale bars, 5 μm. **f** Integrated density of (**e**), Vector, *n* = 67; MFN1-FLAG, *n* = 73; MFN2-FLAG, *n* = 72. ***p < 0.001. Mean with SD.

To confirm the presence of PerMit sites, we used a bimolecular fluorescence complementation system^22^. The yellow fluorescent protein variant Venus is split into two fragments. The N terminal fragment of Venus is either tagged with HA for cytosolic expression or fused to the C terminus of Tom20 for mitochondrial outer membrane expression. The C terminal fragment of Venus is tagged with Myc and fused to the transmembrane region of PEX26 for peroxisomal membrane expression (Fig. 4a). The expression of each fragment was validated by western blots (Fig. 4b). The localization of each fragment was confirmed by immunofluorescent staining of HA or Myc tag. Po-V(C) colocalizes with peroxisome protein ABCD3, while Mito-V(N) colocalizes with mitochondrial protein COX4 (Fig. 4c). We generated two HeLa cell lines, one stably expressing Cyto-V(N) and Po-V(C) (control) and another stably expressing Mito-V(N) and Po-V(C) (PerMit Venus). The fluorescent signal of Venus can only be detected in PerMit Venus but not the control cells (Fig. 4d). Mitofusins were expressed at different levels to induce either mitochondria clustering (Fig. 4e, f) or elongation (Supplementary Fig. 3) in PerMit Venus cells. In the presence of MFN1-Flag or MFN2-Flag, the intensity and the numbers of PerMit Venus sites were significantly increased (Fig. 4e, f and Supplementary Fig. 3).

### Endogenous mitofusins locate on the PerMit contacting sites

Mitofusins are known to locate on the mitochondria surface to bridge the association of two mitochondria^23^. It also functions as one of the bridging molecules on mitochondria to associate with ER^24^. Since mitochondria and peroxisomes cluster upon MFN induction, we wonder if mitofusins also bridge the contact between mitochondria and peroxisomes. In mitofusins overexpressed conditions, the clustering is overwhelming that no clear contacting site can be defined. Therefore, we examined the location of endogenous MFN1 and MFN2.

**Fig. 5:**
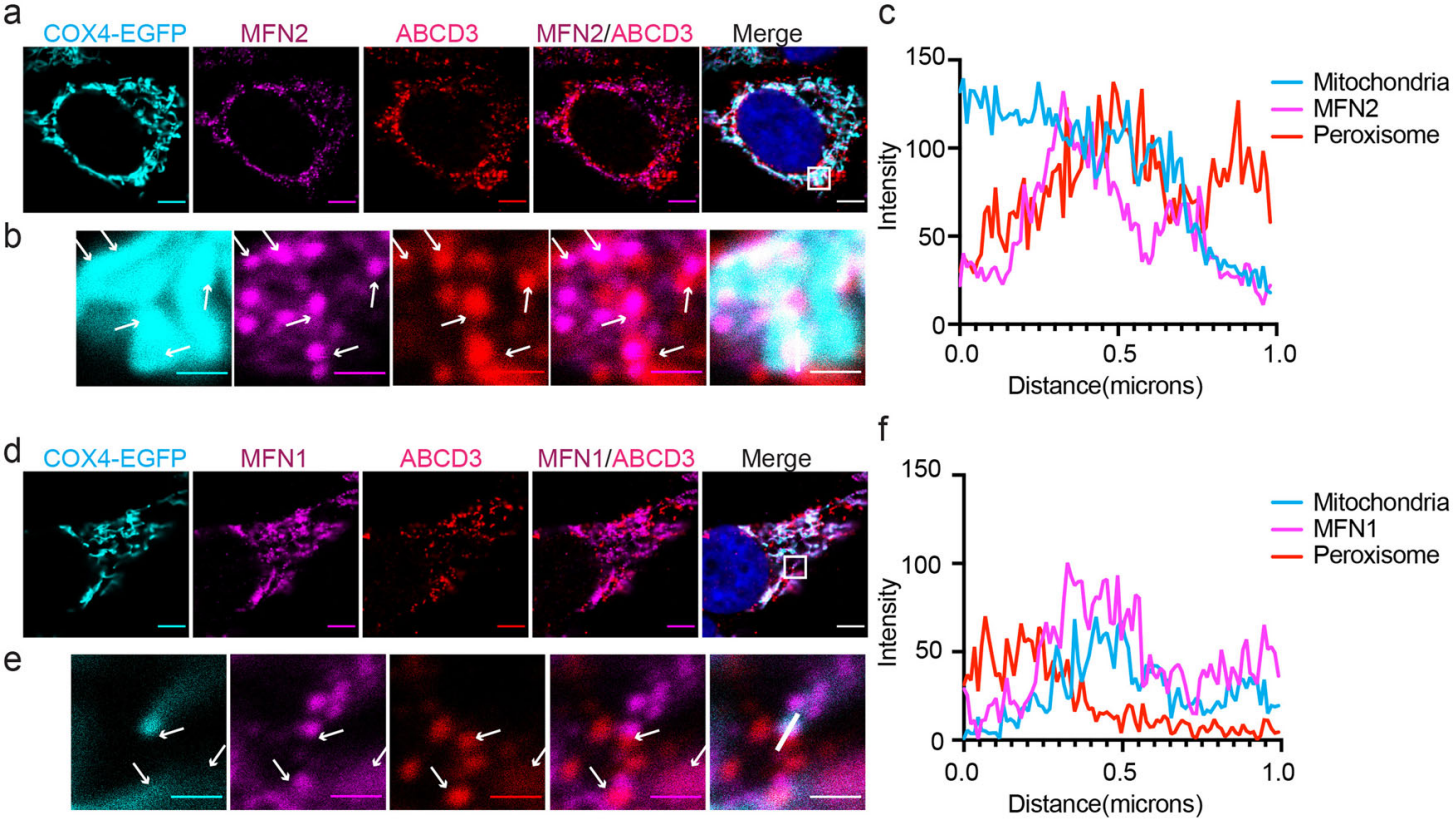
Endogenous mitofusins locate on the mitochondrion-peroxisome contacting sites. **a** Immunofluorescence images of endogenous MFN2. Confocal microscopy analysis of HeLa cells expressing COX4-EGFP (mitochondria), immunostained for MFN2 (Alexa Fluor 555) and peroxisomal protein ABCD3 (Alexa Fluor 647). Scale bars: 5 μm. **b** The zoomed-in images of the white box in (**a**). White arrows point the places where ABCD3 overlaps with MFN. Scale bars: 1 μm. **c** Histograms display measured fluorescence intensity along the white line in the merge panels in **b**, with the cyan line represents mitochondria, the magenta line represents endogenous MFN2, and the red line represents peroxisome. **d**–**f** Immunofluorescence images and analysis of endogenous MFN1. Similar experiments were performed as in (**a**–**c**).

First, we generated a stable HeLa cell line expressing mitochondrial marker COX4-EGFP. Peroxisomal ABCD3 was stained to mark peroxisomes. MFN1 and MFN2 were also stained with specific antibodies to highlight their relative location. Comparing with mitochondrial matrix protein COX4-EGFP (cyan), mitofusins (magenta) are not evenly distributed on the mitochondrial surface (Fig. 5a). Interestingly, in some areas where MFN2 are enriched, there are also strong ABCD3 signals, indicating potential organelle contacting at those spots (Fig. 5b). Fluorescence intensity quantification also confirms these observations (Fig. 5c). Similar results can also be observed with MFN1 (Fig. 5d–f).

### Dominant-negative MFN2 truncation blocks PerMit clustering

**Fig. 6:**
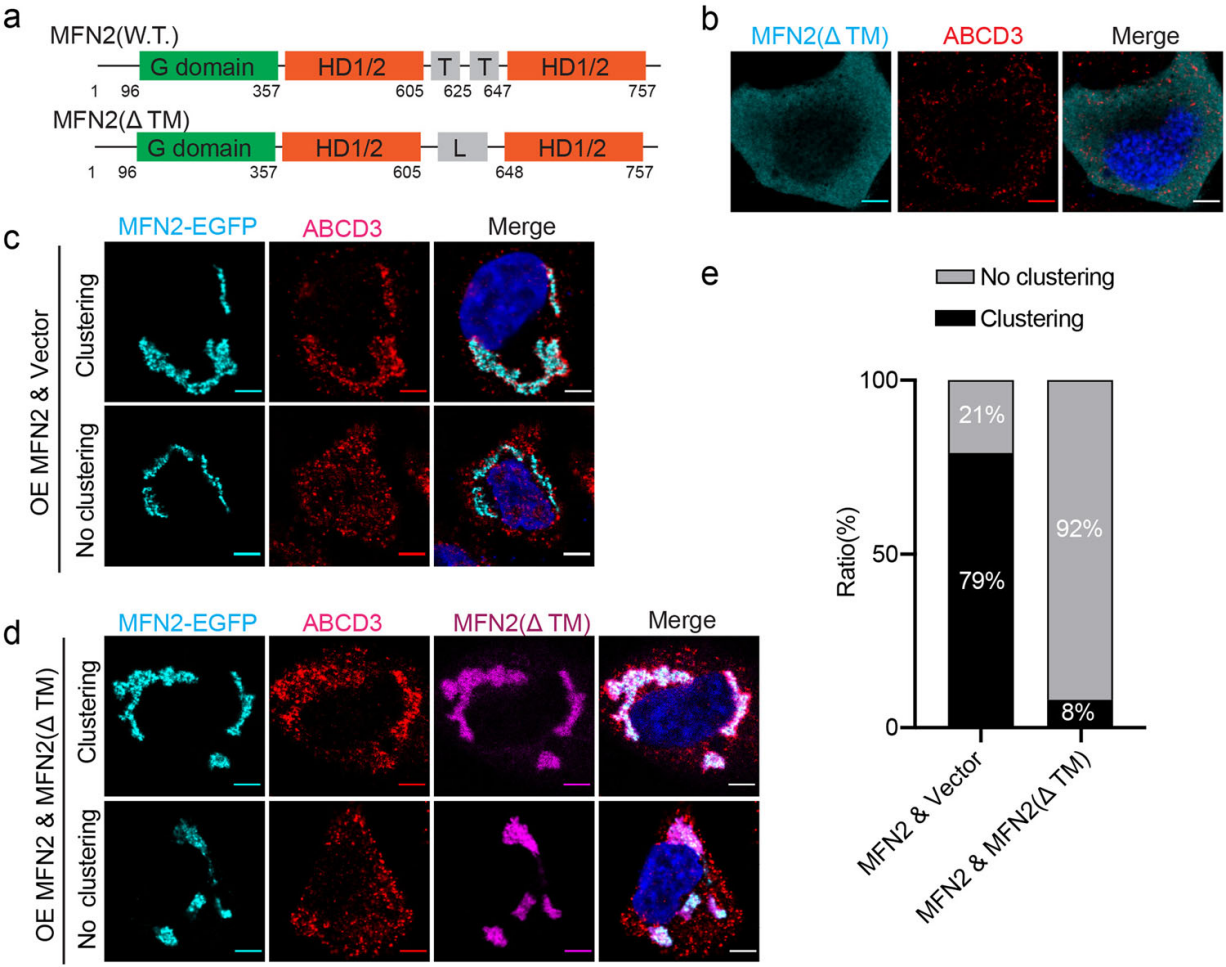
MFN2(ΔTM) blocks mitochondrion-peroxisome clustering. **a** Schematic of the organization of wild type MFN2 and MFN2(ΔTM). G domain, GTPase domain; HD1/2, helical domain 1/2; T, transmembrane region; L, a Gly-Ser linker. Amino acids 606–647 in wild-type MFN2 were replaced with a 10 amino acid linker GGSGGGSGGG. **b** Confocal micrographs of HeLa cells transfected with FLAG-tagged MFN2(ΔTM). Cells were immunostained for endogenous ABCD3 (Alexa Fluor 647) and FLAG (Alexa Fluor 555). Representative confocal micrographs of HeLa cells transfected with (**d**) or without (**c**) MFN2(ΔTM)-FLAG. MFN2-EGFP was overexpressed to induce mitochondrion-peroxisome clustering in both conditions. Cells were stained for ABCD3 and MFN2(ΔTM)-FLAG. **e** The statistics of cell numbers in different stages were analyzed for experiments in **c** (*n* = 240 cells) and **d** (*n* = 106 cells).

MFN2 is a GTPase, which composes a GTPase domain (G domain) at the N terminus, followed by a helical domain (HD), trans-membranes (TMs), and another HD domain^25^. It was proposed that mitofusins mediate mitochondria fusion by homo- or hetero-dimerization via G domain while the TMs anchor on each mitochondrion^26,27^. We wonder if PerMit clustering is formed based on the same mechanism. However, it’s technically challenging to distinguish the peroxisomal localized mitofusins. Therefore, we generated an MFN2 truncation (MFN2ΔTM), where the TM regions (residues 605–647) are replaced with a linker containing 2.5 repeats of GGSG (Fig. 6a). Unlike wild-type MFN2 mainly locates on mitochondria, MFN2(ΔTM) has an even cytosolic distribution (Fig. 6b). In wild-type HeLa cells, overexpression of MFN2-EGFP causes more than 70% of cells to show the clustered mitochondria and peroxisomes (Fig. 6c, e). However, when MFN2(ΔTM) is co-expressed with MFN2-EGFP, the percentage of cells with clustered mitochondria and peroxisomes is significantly reduced (Fig. 6d, e). Interestingly, MFN2(ΔTM) is also recruited to mitochondria. It does not affect mitochondria fusion but significantly inhibits peroxisome clustering (Fig. 6d). Overall, MFN2(ΔTM) functions as the dominant-negative MFN2 mutant to inhibit PerMit clustering, possibly through binding and inhibiting wild-type MFN2.

### The number of PerMit contacting sites is increased upon MFNs upregulation

**Fig. 7:**
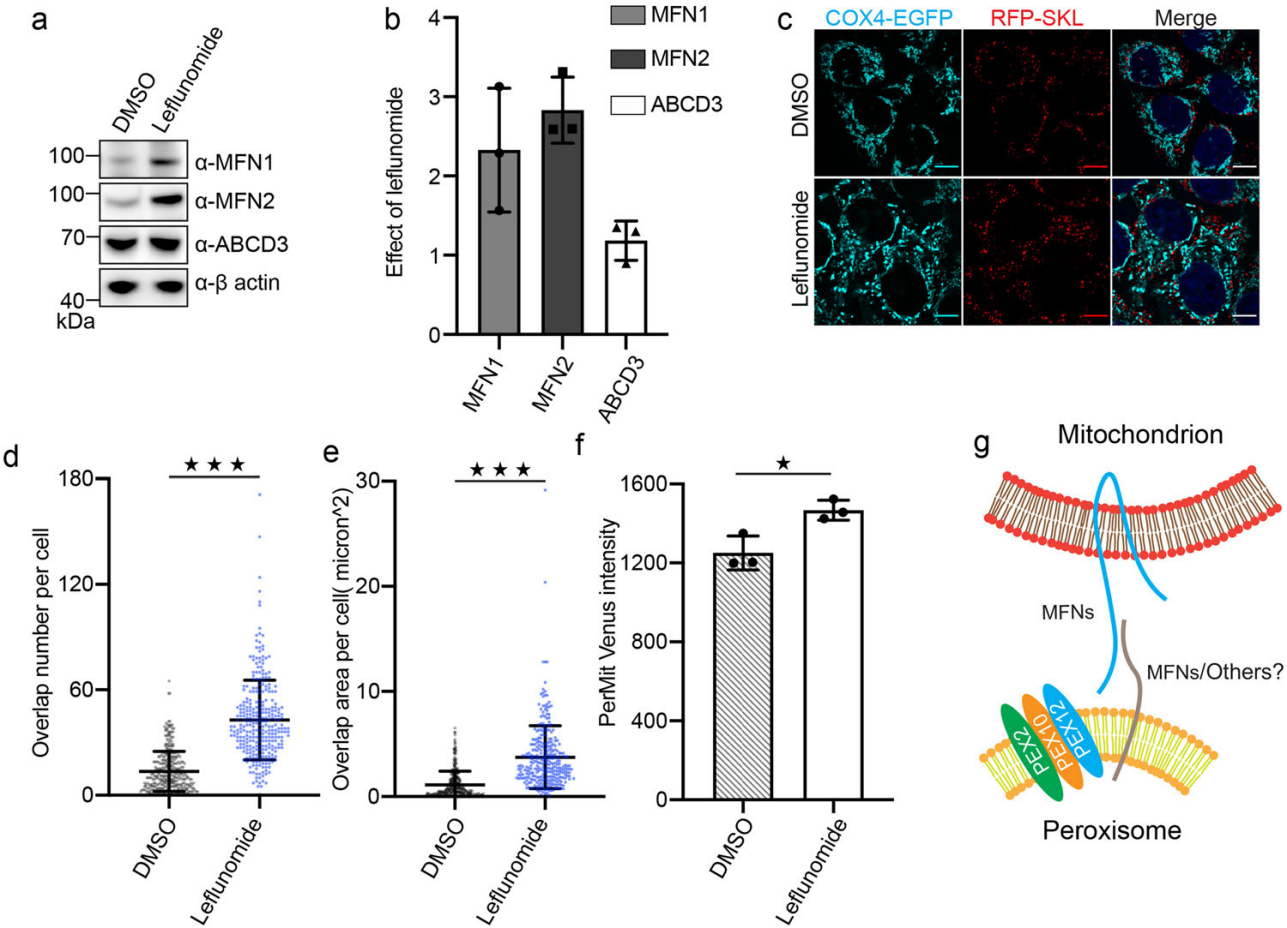
Upregulation of endogenous mitofusins stimulates mitochondrion-peroxisome contacting. **a** Immuno-blots of MFN1, MFN2, and ABCD3 in cells treated with/without leflunomide. **b** Fold change of protein levels on immune-blots (**a**), quantified with ImageJ (*n* = 3, error bars represent standard deviation). **c** Representative fluorescence images of the HeLa cells stably expressing COX4-EGFP and RFP-SKL, treated with either DMSO or leflunomide (50 μM, 48 h). **d**, **e** Quantification of peroxisome and mitochondrion overlap in images collected in (**c**). Scatter dot plots showing peroxisome and mitochondria overlap number and area in HeLa cells incubated with DMSO (*n* = 348 cells) and 50 μM leflunomide (*n* = 310 cells) respectively for 48 h. ***p < 0.001. Mean with SD. *P* values calculated via unpaired Student’s t-test (two-tailed). **f** The Venus fluorescence intensity in cells treated with either DMSO or 50 μM leflunomide were analyzed with flow cytometry. At each condition, the mean fluorescence intensity (MFI) is calculated from 20,000 to 60,000 cells. The experiment was repeated for three times. The columns represent average MFI (mean fluorescence intensity) with SD. *p < 0.05, calculated via unpaired Student’s t-test (two-tailed). **g** A working model for MFN mediated PerMit contacting.

There are limited numbers of PerMit contacting sites under normal conditions, but the overexpressed mitofusin-EGFP induces significant PerMit clustering in cells (Figs. 3 and 4). To examine if the upregulation of endogenous mitofusins would cause a similar effect as shown with overexpressed mitofusins, we treated cells with leflunomide. Leflunomide is a drug approved to treat adult rheumatoid arthritis, and it has been reported to induce mitofusin expression in cells^28^. We treated HeLa cells with 50 μM leflunomide for 48 h, and the protein levels of MFN1 and MFN2 were significantly increased more than two folds comparing with the loading control actin (Fig. 7a, b). To further mapping the clustering status of mitochondria and peroxisomes, a stable cell line expressing both the mitochondrial marker COX4-EGFP and the peroxisomal marker RFP-SKL was generated (Fig. 7c). In the presence of leflunomide, the number of EGFP/RFP overlapping sites and the area of overlapping regions of EGFP/RFP both increase (Fig. 7d, e). These results indicate that when the endogenous mitofusin expression is induced, there are increased PerMit contacts in cells. To further confirm the changes at PerMit sites, we used proximity-based BiFC assay to analyze Venus signals with or without leflunomide (Fig. 7f). Flow cytometry analysis show upregulated Venus intensity in leflunomide treated cells, suggesting the increase of PerMit contacts.

## Discussion

Mapping organelle interactions are essential to systematically understand the regulation of cellular metabolism and signaling. Before addressing the functional relationship between different organelles, understanding the key protein components contributing to organelle contact is essential but challenging. Here we used the PUP-IT proximity tagging approach to label proteins in proximity of peroxisomes and identified peroxisome regulators on mitochondria. PUP-IT is a well-suited method to identify organelle contacting sites since it captures weak and transient interactions and has been validated to be used for various proteins^17,29–32^.

In addition to MFNs, a few other mitochondrial proteins have been identified with PUP-IT. Although PafA fused PEX proteins seem to exclusively locate on peroxisomes in this study, others have reported mitochondrial localization of peroxisomal proteins in peroxisome deficient cells^33^. PEX2, PEX10, and PEX12 are ubiquitin ligases, but MFN level does not change significantly with overexpressed PEX10 or PEX12 (Fig. 2). It would be interesting to see if PEX10/12 interacts with other mitochondria proteins or targets them for ubiquitination and degradation.

The main discovery of this study is that mitofusins reside in close proximity to peroxisomal proteins, and contribute to the co-clustering of mitochondria and peroxisomes. The formation of mitochondrial clustering by mitofusin overexpression has been observed before^11^. However, the correlation of this type of clustering with other organelles has not been revealed yet. We found peroxisomes are the only co-clustering organelles among four other organelles examined in this study. Furthermore, we also observed uneven mitochondrial surface mitofusin distribution is correlated with PerMit contacting in wild-type cells, suggesting potential roles of mitofusins in regulating PerMit contacting sites and peroxisome cellular distribution.

The exact mechanism of mitofusin-mediated PerMit clustering is not known. Our data synergizes with previous findings and suggests a model for mitofusin-mediated organelle contacting (Fig. 7g). In yeast, systematic mapping of PerMit contacts by split fluorophores has revealed Fzo1 (yeast mitofusin) and Pex34 as some of the tether proteins^5^. When full-length Fzo1 is overexpressed, mitochondria are hyper-fused, and Fzo1 is localized to mitochondria-peroxisomes contacting sites. Using a totally different approach in mammalian cells, we also identified mitofusins as potential tether proteins between mitochondria and peroxisomes. First, it is enriched at the PerMit contacting sites. Secondly, the overexpressed cytosolic version of MFN2 blocks PerMit clustering, wherein MFN2(ΔTM) likely functions as a dominant-negative mutant that inhibits wild-type MFN2. More interestingly, the mitochondria clustering is not blocked to the same extent, suggesting mitochondria clustering and peroxisome clustering may rely on two different sets of molecular machinery.

MFN2 is previously reported to be located on both mitochondria and ER to mediate the tethering of these two organelles^24^. Interestingly, some evidence has also been provided that mitochondria and peroxisomes are tethered under physiological conditions^34^. Evaluating colocalization of endogenous MFN and peroxisomes in Fig. 5 is still susceptible to the limits of resolution since an overlap in fluorescence does not necessarily indicate colocalization of two proteins in the same cellular structure. However, the observation of PerMit inhibition by a dominant-negative MFN2 mutant (Fig. 6) increases the confidence that MFN mediates PerMit. In future studies, super-resolution microscopy and electron microscopy will be helpful to fully address the exact localization of MFNs.

Mitofusins are known to mediate organelle fusion via homo- or heterodimerization. It is tempting to speculate that MFNs also locate on peroxisomes to dimerize with mitochondrial MFNs to mediate PerMit clustering. To briefly test this hypothesis, we fused MFN(ΔTM) with either mitochondrial targeting Tom20 or peroxisomal targeting PEX26 (237-305). Immunofluorescence confirmed the organelle-specific localization of PEX26(237-305) and Tom20 (Supplementary Fig. 4a). We found peroxisome-targeting MFNs can induce peroxisome clustering, but not PerMit clustering (Supplementary Fig. 4b, c). On the other hand, mitochondrial targeting MFNs, especially MFN2, is sufficient to induce PerMit clustering (Supplementary Fig. 4c). It seems PerMit clustering is mainly induced by mitochondrial MFNs.

It is always technically challenging to study organelle contact sites. Here we used either overexpression of MFN or leflunomide induction to induce PerMit. Overexpression may exaggerate the effects, and leflunomide may have other effects on cells in addition to inducing mitofusins. We attempted to knock down MFN1 or MFN2 individually. No obvious changes in Split-Venus signal were observed when one of the mitofusins was knocked down. This may be the result of the redundant function for MFN1 and MFN2. Alternatively, MFNs may promote PerMit in certain conditions. Organelle contacts can be induced at certain circumstances and possibly regulated by different factors. ECI2 and Pex34 are also reported to mediate PerMit^4,5^. It is possible there are many factors involved in PerMit at different conditions. Future studies will be required to understand how mitofusins regulate PerMit in different physiologically related conditions. It would also be interesting to know how mitofusins cooperate with other protein partners at the mitochondria-peroxisomes contacting sites, whether mitofusins regulate the PerMit metabolic cooperation for the β-oxidation of fatty acids, ROS homeostasis, or anti-viral signaling. In addition, MFN2 mutations closely correlate with peripheral neuropathy, such as Charcot-Marie-Tooth type 2 A (CMT2A)^35^. The pathology is contributed partly by defected organelle contacting. Defective peroxisomal functions are also reported in various neuropathy^36^. It would also be interesting to address the role of PerMit contacts under a disease-relevant condition.

## Methods

### Antibodies

Detecting reagents used for western blotting include: streptavidin-HRP (Cell Signaling, 3999S, 1:3000), anti-Myc (Cell Signaling, 2276S, 1:3000), anti-V5 (Abcam, ab27671, 1:3000), anti-β actin (GenScript, A00702, 1:3,000), anti-FLAG (GNI, GNI14110-FG, 1:3000), anti-ABCD3 (Sigma, P0497, 1:3000), anti-MFN1 (Cell Signaling, D6E2S, 1:3000), anti-MFN2 (Cell Signaling, D1E9, 1:3000). Antibodies used for immunofluorescence include: anti-MFN1 (Cell Signaling, D6E2S, 1:500), anti-MFN2 (Cell Signaling, D1E9, 1:500), anti-calnexin (Abcam, ab22595, 1:500), anti-EEA1 (Cell Signaling, C45B10, 1:500), anti-GM130 (Abcam, ab52649, 1:500), anti-LAMP1 (Cell Signaling, D2D11, 1:500), anti-ABCD3 (Sigma, SAB4200181, 1:500), anti-ABCD3 (Sigma, P0497, 1:500), anti-catalase (Merck/Millipore, 219010, 1:500), anti-FLAG (GNI, GNI14110-FG, 1:500), anti-FLAG (Proteintech, 20543-1-AP, 1:500), anti-Myc (Cell Signaling, 2276S, 1:500), anti-COX4 (Proteintech, 11242-1-AP, 1:500).

### Molecular cloning

Human *pex2*, *pex10*, and *pex12* cDNAs were obtained from Jiahuai Han’s lab. *pafA* gene from *Corynebacterium glutamicum* was synthesized by Qinglan Biotech (Wuxi, China). *pafA* was genetically fused to different *pex* genes by overlapping PCR and individually subcloned into the lentiviral vector at the *BamH1* restriction enzyme cleavage site by Gibson cloning. BCCP-PupE-IRES-BFP was cloned into the expression plasmid of the Tet-On 3G inducible expression system (Clontech cat#: 631168). Other genetic fusions, such as MFN1-EGFP, MFN2-EGFP, and MFN2(ΔTM), were generated by overlapping PCR and cloned into pCDNA3.1 at the BamH1 site. RFP-SKL, COX4-EGFP were cloned into pHR_EF1α at the *BamH1* restriction enzyme cleavage site by Gibson cloning. All constructs were verified by DNA sequencing.

### Cell culture and transfection

HeLa cells (ATCC, CCL-2) and 293T (ATCC, CRL-1573) cells were maintained in a cell culture incubator at 37 °C with 5% CO_2_. Cells were cultured in DMEM medium (Thermo; C11995500CP) supplemented with 10% FBS (GEMINI; 900-108). Transfection of HeLa cells was performed using the Lipofectamine^®^ 3000 Transfection Reagent (Thermo, L3000015). Transfection steps were performed according to the manufacturer’s instructions. Cells were tested and confirmed negative for mycoplasma.

### Lentivirus generation and infection

RFP-SKL, COX4-EGFP were cloned into pHR_EF1α at the *BamH1* restriction enzyme cleavage site by Gibson cloning. Lentiviral constructs were co-transfected with their respective packaging vectors (pCMV-dR8.91 and pMD2G) into 293T cells in a 1:1:0.1 molar ratio in a six-well cell culture plate. The medium was changed 16 h after transfection (2.5 ml per well for a six-well cell culture plate). The medium was collected 48 h after transfection. Cell debris in the medium was removed via centrifugation (10 min at 1000 × *g*), and lentivirus-containing supernatant was cleared from filtration through a 0.45 µm polyethersulfone membrane (Millipore, SLHV033RB).

### The generation of stable cell lines

To generate the iPUP HeLa cell line, pTet3G-Bio-Pup(E)–IRES– BFP and pTre3G were packed into lentiviruses and then co-transfected into the HeLa cells for BFP-positive cell sorting. Cells proliferated from single clones were analyzed with flow cytometry after three weeks with or without doxycycline induction. The expression of bio-Pup(E) in BFP-positive cells was also confirmed by western blotting.

To generate the HeLa cell line stably expressing mitochondrial and peroxisomal markers, HeLa cells were infected with COX4-EGFP and RFP-SKL lentiviruses. EGFP and RFP double-positive cells were sorted into 96-well plates with one cell per well for single clone selection. COX4-EGFP HeLa cell line was generated in the same way.

### Mass spectrometry and data analysis

PUP-IT assay was performed as previously described^17^. PUP-IT^XIAP^, PUP-IT^PEX2^, PUP-IT^PEX10^, and PUP-IT^PEX12^ cells were grown to 2 × 10^7^ cells. Doxycycline and biotin were added to the medium at 2 μg/ml and 4 μM for 30 h. After labeling, cells were harvested, washed by PBS, and lysed by lysis buffer (50 mM Tris, 200 mM NaCl, 1% NP-40, pH 7.5). Urea was added to the lysate to 8 M. The denatured lysate was sequentially treated with10 mM DTT (Dithiothreitol) at 56 °C for 1 h, 25 mM IAM (Iodoacetamide) protected from light for 45 min, and 40 mM DTT at room temperature for 30 min. 50 µl streptavidin beads (Pierce, 88816) were added to each sample to rotate and incubate for 1 h at room temperature. The beads were washed sequentially by 1 ml buffer 1 (50 mM Tris 8.0, 8 M urea, 200 mM NaCl, 0.2% SDS) for three times, 1 ml buffer 2 (50 mM Tris 8.0, 8 M urea, 200 mM NaCl) for three times, and 1 ml buffer 3 (50 mM Tris 8.0, 0.5 mM EDTA, 1 mM DTT) for two times and 1 ml 50 mM NH_4_HCO_3_ aqueous solution for three times at room temperature. Finally, the on-beads trypsin digestion was performed, and the digested peptides were separated and analyzed on an Easy-nLC 1000 system coupled to a Q Exactive HF mass spectrometer (Thermo Fisher Scientific). The raw data were processed and searched with MaxQuant 1.5.4.1. GGE (K), oxidation (M), acetyl (protein N-terminus), and deamidation (NQ) were set as variable modifications. The FDR of both peptide identification and protein identification is set to be 1%. LFQ was used to quantify the difference in protein abundances between different samples. The mass spectrometry proteomics data have been deposited to the ProteomeXchange Consortium (http://proteomecentral.proteomexchange.org) via the iProX partner repository^37^ with the dataset identifier PXD032661.

For statistical analysis, the R (3.6.0) package Limma (3.48.1) was applied for the analysis of LFQ intensity data as previously reported^38–40^. Data processing includes imputation, log2 transformation, and centralization. Limma-based t-test was used for significant difference testing of data. The candidate proteins were determined by a moderated t-test (*P*-value < 0.05) and fold change (fold change > 1.3). The fold changes of intensity and *P*-values were plotted with log transformation into a volcano plot by Prism. The R package clusterProfiler (DOI: 10.18129/B9. bioc. clusterProfiler) was used to identify peroxisomal proteins and mitochondrial proteins. Uniprot database and MitoCarta3.0 were also used to identify peroxisomal proteins, mitochondrial proteins, and ER proteins.

### Imaging and immunofluorescence

HeLa cells were plated on coverslips and incubated at 37 °C and 5% CO_2_ before staining. After 24 h, cells were washed with PBS three times and fixed with 4% paraformaldehyde in PBS, pH7.4, at room temperature for 15 min, then permeabilized with 0.1% NP-40 in PBS for 15 min and blocked with 2% BSA in cell staining buffer (4A Biotech; FXP005) at room temperature for 1 h. After blocking, the cells were incubated with primary antibodies diluted in blocking buffer at 4 °C overnight. Wash cells and incubate with appropriately fluorochrome-conjugated second antibodies for 1 h at room temperature. After incubation, cells were stained with DAPI for 10 min and mounted with Mounting Medium (Vectorlabs, H-1000). Images were captured by Leica SP8 confocal microscope (objective ×63 1.40 NA OIL).

### Split Venus reporter assay

Venus, a variant of YFP was used for this assay. The chosen human outer mitochondrial membrane protein Tom20 (full length) was genetically fused with N terminus of Venus fluorescent protein (1-154), the transmembrane fragment of peroxisomal membrane protein PEX26 (237-305) was genetically fused with the C terminus of Venus fluorescent protein (155-238). Images were captured by Leica SP8 confocal microscope (objective ×63 1.40 NA OIL).

### Immunoprecipitation and Western Blotting assay

The cells were washed in PBS three times and lysed directly using cell lysis buffer (Beyotime, P0013) supplemented with 100× protease inhibitor cocktail (Biomake, B14001) for 1 h at 4 °C. Lysates were centrifuged at 4 °C at maximum speed (15,000 × *g*) for 10 min. The supernatant was subjected to BCA Protein Assay (Transgen, DQ111-01) to quantify protein concentration.

For immunoprecipitation with V5 antibody, 20 µl protein G beads (Thermo, UH288734) were centrifuged at 4 °C at 500 g for 3 min to remove the supernatant. The beads were resuspended by 50 µl PBS and centrifuged at 4 °C at 500 g for 3 min to remove the supernatant. The beads were resuspended with 100 µl lysis buffer and added 2 µl V5 antibody. The beads and the antibody were incubated on a rotator at 4 °C overnight. Cells were lysed, and the V5 antibody-conjugated beads were added to the cell lysates to incubate at 4 °C for 3 h. The beads were pelleted and washed with lysis buffer for three times and were resuspended in 1× denaturing loading buffer. The beads were heated at 95 °C for 10 min and centrifuged at 12,000 rpm for 2 min before SDS–PAGE analysis. The supernatant was loaded on a 4–20% Bis-Tris SDS-PAGE gel (Genescript, M00656), transferred to PVDF membranes (Millipore, IPVH00010), and probed with specific antibodies. Western blot Images were quantified with ImageJ. The frame region containing a protein band was defined a selection frame. The pixel density of each band was measured with imageJ. Protein levels were normalized to β-actin.

### Image quantification and statistical analysis

To quantify the overlap between peroxisomes and mitochondria, the COX4-EGFP channel and RFP-SKL channel were set a threshold by the ImageJ software, and the image analysis command “AND” was run to obtain the overlap image of the COX4-EGFP channel and RFP-SKL channel. “Watershed” was applied, and “Analyze Particles” was used to obtain the number and area of the double-positive signal of peroxisome and mitochondria. Quantitative data are presented as mean ± SD (Standard Deviation). Statistical significance was assessed on the basis of *P* values calculated via unpaired Student’s t-test (two-tailed) in either Prism or Excel. The number of experiments (*n*) used for statistical evaluation is specified in relevant figure legends.

### Statistics and reproducibility

Western blots of endogenous proteins were quantified using ImageJ software. Data were analyzed and graphed using Prism 8 and Excel. All data are represented as the mean ± SD. Unpaired two-tailed Student’s t-test was used for statistical significance. In each sample, the number of cells used for statistics range from 50 to 400. All the experiments were repeated 2–4 times.

### Reporting summary

Further information on research design is available in the Nature Research Reporting Summary linked to this article.

## Supplementary information

Supplementary Information

Description of Additional Supplementary Files

Supplementary Data 1

Supplementary Data 2

Reporting Summary

## Data Availability

All pertinent data are available within the manuscript or upon request. Source data for graphs in the figure are provided as Supplementary Data 1 and 2. Uncropped gel images are included in Supplementary Figs. 5 and 6. The mass spectrometry proteomics data are available at ProteomeXchange Consortium (http://proteomecentral.proteomexchange.org) via the iProX partner repository with the dataset identifier IPX0003347000/PXD032661.
