# Supplementary material for: The MFN1 and MFN2 mitofusins promote clustering between mitochondria and peroxisomes
Source: Commun Biol. 2022 May 6;5:423. doi: 10.1038/s42003-022-03377-x (PMC9076876; doi:10.1038/s42003-022-03377-x)
Supplement: Supplementary file 1 — Supplementary Information [file 42003_2022_3377_MOESM1_ESM.pdf]

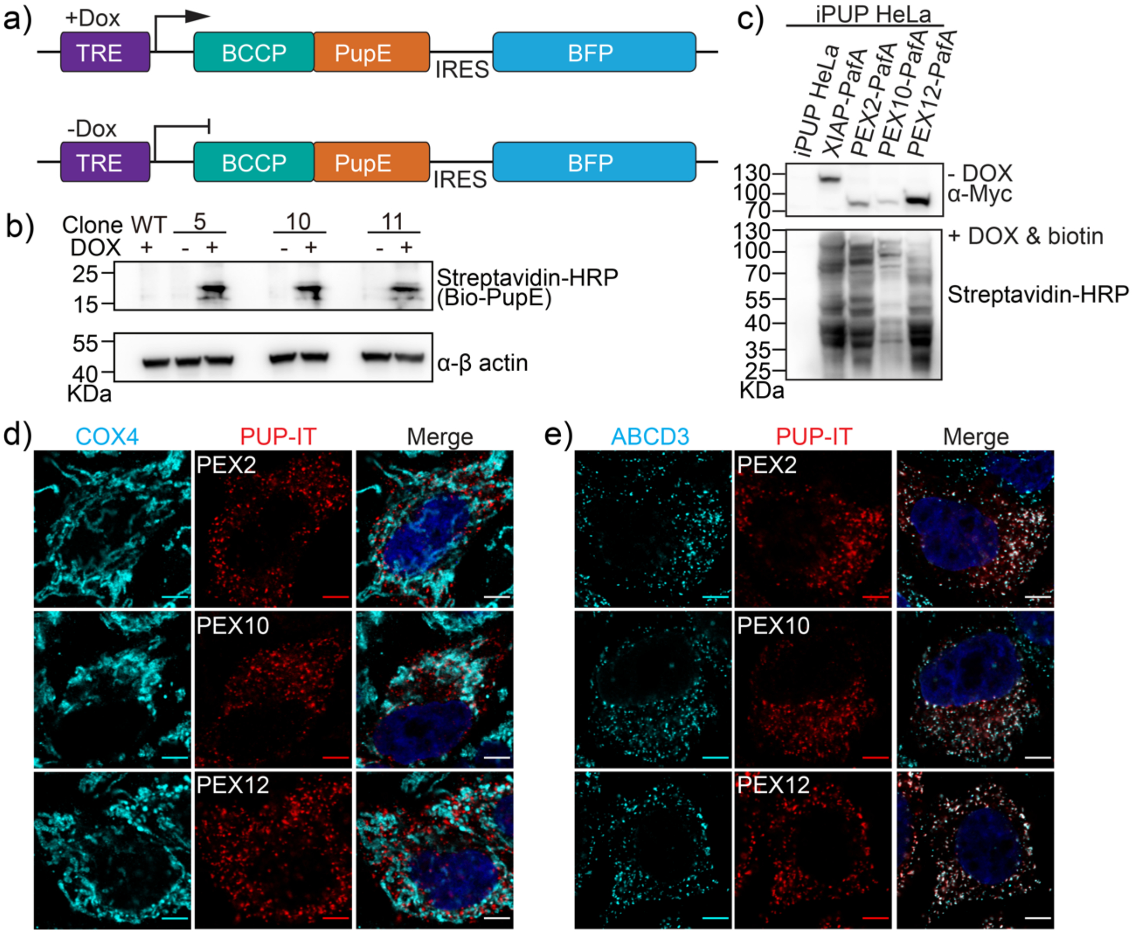


**Supplementary Figure 1. Generation and validation of the PUP-IT system.**
**a**, Schematics for the design of induced PupE expression in iPUP cell line. BCCP domain fused PupE was cloned under the TET-ON promoter with the blue fluorescent protein (BFP) separated with an internal ribosome entry site (IRES). The addition of Doxycycline (Dox) induces BCCP-PupE (bio-PupE) expression. **b**, Western blots examining bio-PupE expression in different single-cell clones in the presence of Dox. **c**, iPUP HeLa cells were transfected with Myc tagged XIAP-PafA, PEX2-PafA, PEX10-PafA, or PEX12-PafA, the expression of PafA fusions were examined with anti-Myc immuno-blot, and the extent of bio-PupE labeling was detected with streptavidin-HRP. **d, e,** Immunofluorescence images of different PafA fusions. Cells were stained for Myc (PUP-IT), COX4 (mitochondria), and ABCD3 (peroxisomes).

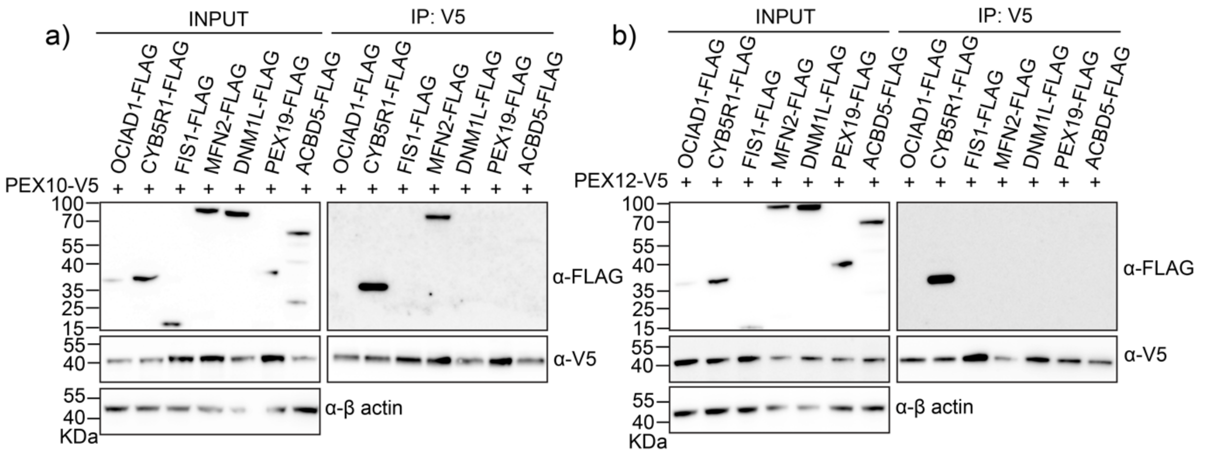


**Supplementary Figure 2. Biochemical validation for PEX10 and PEX12 interacting proteins.**

**a**, Co-immunoprecipitation of PEX10 and interacting candidates. V5 tagged PEX10 was co-transfected with different FLAG-tagged interacting candidates, including OCIAD1, FIS1, DNM1L, PEX19, and ACBD5, in HEK293T cells. PEX10-V5 were immunoprecipitated and immunoblots were performed with indicated antibodies. **b**, Co-immunoprecipitation of PEX12 and interacting candidates.

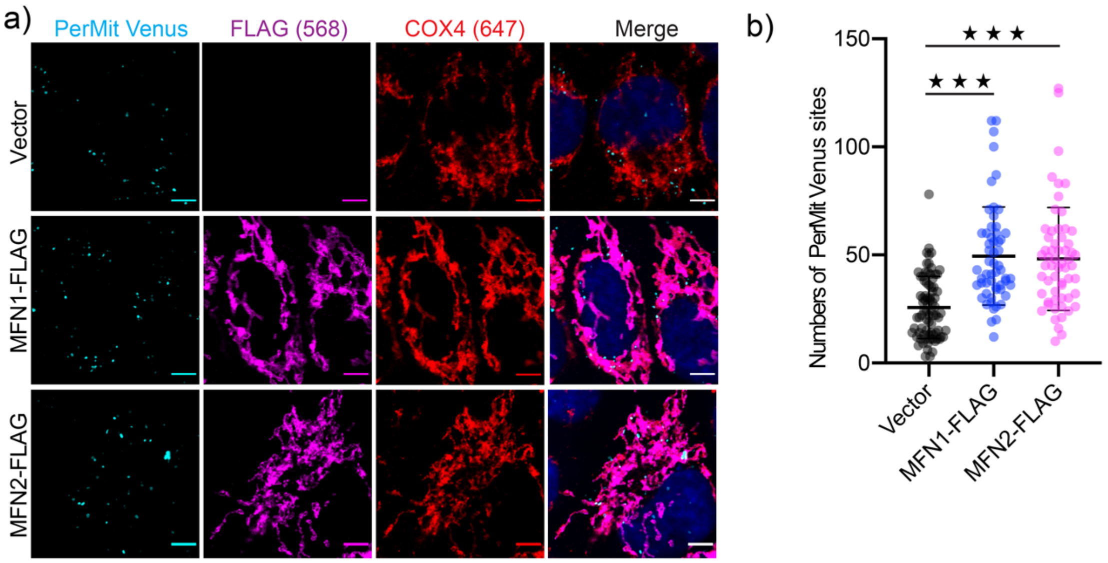


**Supplementary Figure 3. Supplementary to Fig. 4e and f.**

**a,** HeLa cells were transfected with Vector, MFN1-FLAG or MFN2-FLAG plasmids (0.5 μg DNA/35 mm cell culture dish) for 6-12 hrs to induce mitochondria elongation. FLAG (Alexa Fluor 568) and COX4 (Alexa Fluor 647) were immunostained. Scale bars, 5 μm. **b,** The statistics of PerMit Venus sites in (a), Vector, n = 80 cells, MFN1-FLAG, n = 57 cells, MFN2-FLAG, n = 59 cells. ***$p<0.001$. Mean with SD. P values calculated via unpaired Student's t-test (two-tailed).

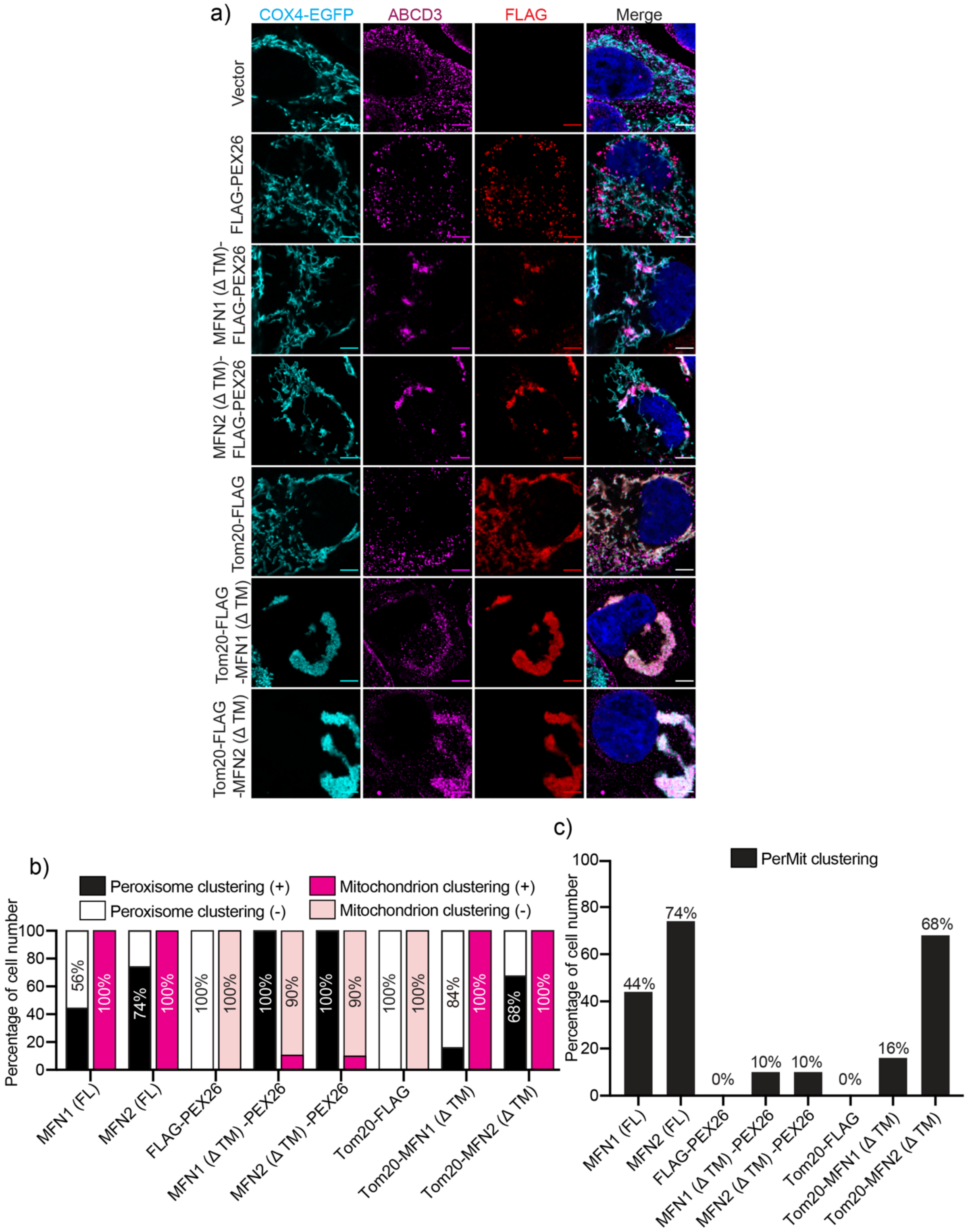


**Supplementary Figure 4. PerMit co-clustering are mainly induced by mitochondrial MFNs.**

**a**, Immunofluorescence images of mitochondria and peroxisomes with different localized MFNs. HeLa cells were transfected with empty vector, FLAG-PEX26(237-305), MFN1 (ΔTM)-FLAG-PEX26, MFN2 (ΔTM)-FLAG-PEX26, Tom20-FLAG, Tom20-FLAG-MFN1 (Δ TM), or Tom20-FLAG-MFN2 (ΔTM) plasmids for 36 hrs. Peroxisomal membrane protein ABCD3

(Alexa Fluor 555) and FLAG tag (Alexa Fluor 647) were immunostained. Scale bars, 5 μm. **b,c**, The statistics for the percentage of cells with different organelle clustering status for the experiment in (**a**). Cells were transfected with indicated plasmids. Total cell numbers used for the analysis are 124, 159, 106, 86, 110, 97, 137 and 207 accordingly from left to right.

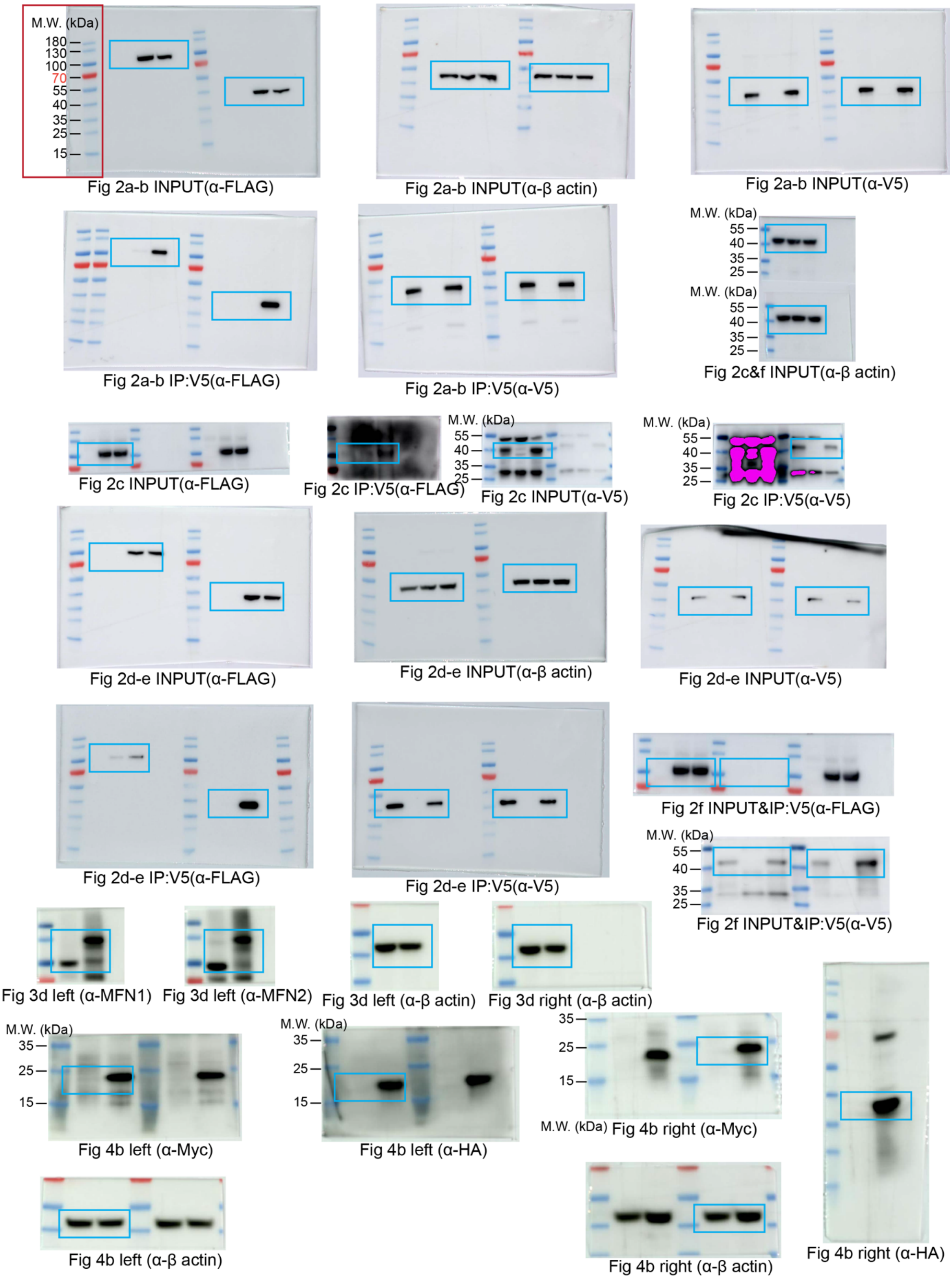


**Supplementary Figure 5. Uncropped original scans of blots.** The same protein standards were used for all studies and the gel images was obtained with Amersham Imager 600 or BioRad ChemiDoc. The red box highlights the molecular weight of each protein standard. In blots where the orange marker protein (70 kDa) is not shown, additional labels are added. The blue boxes highlight the cropped region and related figures are marked under each image.

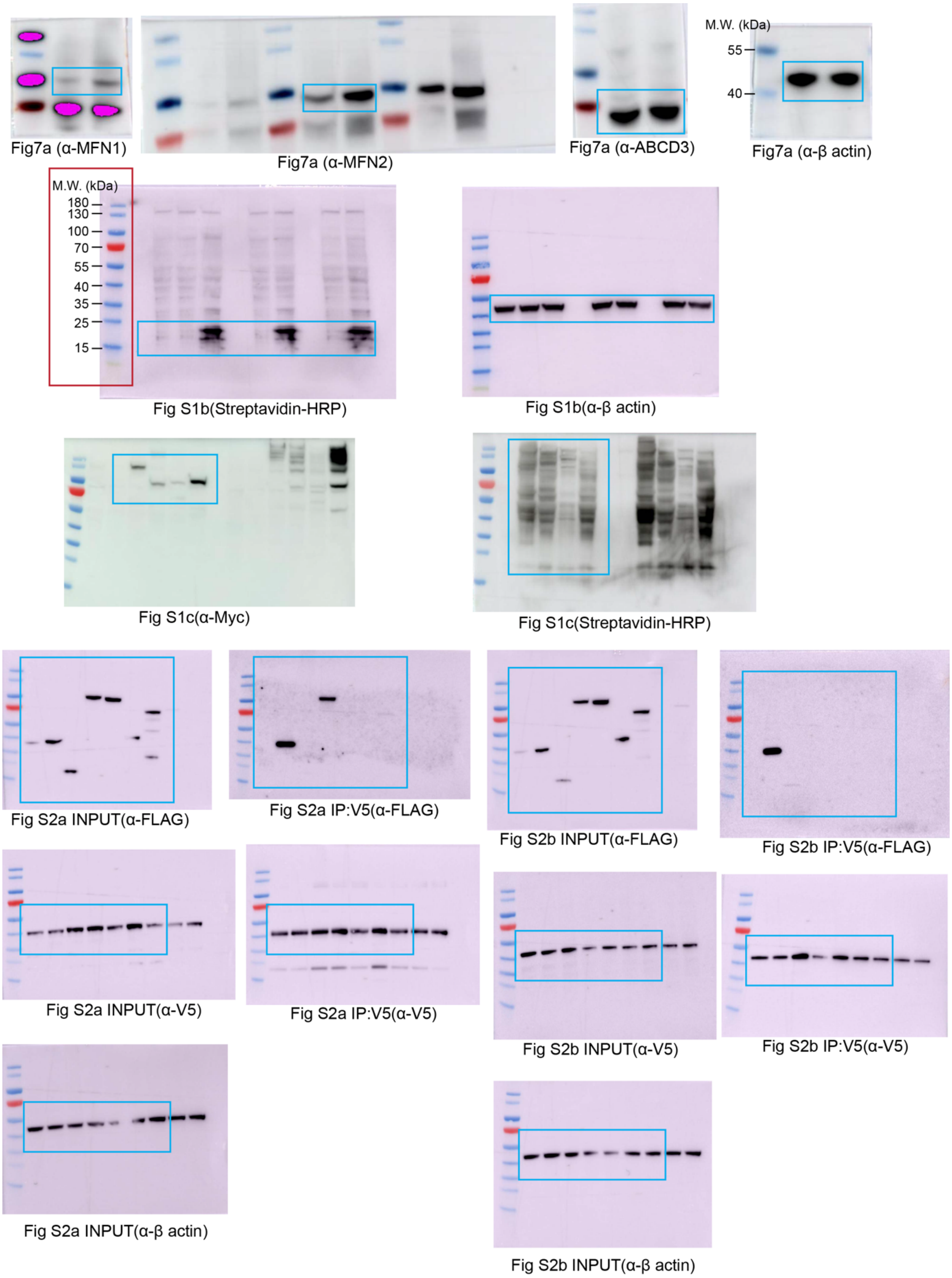


**Supplementary Figure 6. Uncropped original scans of blots (continued).** The same protein standards were used for all studies. The red box highlights the molecular weight of each protein standard. The blue boxes highlight the cropped region and related figures are marked under each image.
