# Supplementary material for: The MFN1 and MFN2 mitofusins promote clustering between mitochondria and peroxisomes
Source: Commun Biol. 2022 May 6;5:423. doi: 10.1038/s42003-022-03377-x (PMC9076876; doi:10.1038/s42003-022-03377-x)
Supplement: Supplementary file 2 — Description of Additional Supplementary Files [file 42003_2022_3377_MOESM2_ESM.pdf]

Description of Additional Supplementary Files

File name: Supplementary Data 1
Description: Mass spec analysis and source data for Fig 1c-e.

File name: Supplementary Data 2
Description: The source data for Fig 4f, 5c, 5f, 6e, 7b, and 7d-f
