# Supplementary material for: The MFN1 and MFN2 mitofusins promote clustering between mitochondria and peroxisomes
Source: Commun Biol. 2022 May 6;5:423. doi: 10.1038/s42003-022-03377-x (PMC9076876; doi:10.1038/s42003-022-03377-x)
Supplement: Supplementary file 5 — Reporting Summary [file 42003_2022_3377_MOESM5_ESM.pdf]

nature portfolio

Corresponding author(s): Min Zhuang
Last updated by author(s): Yinbo Huo

Reporting Summary

Nature Portfolio wishes to improve the reproducibility of the work that we publish. This form provides structure for consistency and transparency in reporting. For further information on Nature Portfolio policies, see our Editorial Policies and the Editorial Policy Checklist.

Statistics

For all statistical analyses, confirm that the following items are present in the figure legend, table legend, main text, or Methods section.

n/a	Confirmed	
☐	☒	The exact sample size (*n*) for each experimental group/condition, given as a discrete number and unit of measurement
☐	☒	A statement on whether measurements were taken from distinct samples or whether the same sample was measured repeatedly
☐	☒	The statistical test(s) used AND whether they are one- or two-sided<br>*Only common tests should be described solely by name; describe more complex techniques in the Methods section.*
☒	☐	A description of all covariates tested
☒	☐	A description of any assumptions or corrections, such as tests of normality and adjustment for multiple comparisons
☐	☒	A full description of the statistical parameters including central tendency (e.g. means) or other basic estimates (e.g. regression coefficient) AND variation (e.g. standard deviation) or associated estimates of uncertainty (e.g. confidence intervals)
☐	☒	For null hypothesis testing, the test statistic (e.g. *F*, *t*, *r*) with confidence intervals, effect sizes, degrees of freedom and *P* value noted<br>*Give P values as exact values whenever suitable.*
☒	☐	For Bayesian analysis, information on the choice of priors and Markov chain Monte Carlo settings
☒	☐	For hierarchical and complex designs, identification of the appropriate level for tests and full reporting of outcomes
☒	☐	Estimates of effect sizes (e.g. Cohen's *d*, Pearson's *r*), indicating how they were calculated

Our web collection on statistics for biologists contains articles on many of the points above.

Software and code

Policy information about availability of computer code

Data collection	The Mass spectrometry raw data were processed and searched with MaxQuant 1.5.4.1.
Data analysis	The R (3.6.0) package Limma (3.48.1) was applied for the analysis of LFQ intensity data; The R package clusterProfiler (DOI: 10.18129/B9. bioc. clusterProfiler) was used to identify peroxisomal proteins and mitochondrial proteins; ImageJ was used to quantify band intensity in immunoblots; GraphPad Prism 8 was used to plot dot and column plots; Statistical significance was assessed on the basis of P values calculated via unpaired Student's t-test (two-tailed) in Excel; Adobe Illustrator CC2017 was used to demonstrate data.

For manuscripts utilizing custom algorithms or software that are central to the research but not yet described in published literature, software must be made available to editors and reviewers. We strongly encourage code deposition in a community repository (e.g. GitHub). See the Nature Portfolio guidelines for submitting code & software for further information.

Data

Policy information about availability of data

All manuscripts must include a data availability statement. This statement should provide the following information, where applicable:

- Accession codes, unique identifiers, or web links for publicly available datasets
- A description of any restrictions on data availability
- For clinical datasets or third party data, please ensure that the statement adheres to our policy

All pertinent data are available within the manuscript or upon request. All potential interacting proteins that identified by PUP-IT(PEX2/10/12) are available in Supplementary data 1. The uncropped gel images are in supplementary Fig 5. The source data related to the main figures are grouped in supplementary data 2. The mass spectrometry proteomics data are available at ProteomeXchange Consortium (http://proteomecentral.proteomexchange.org) via the iProX partner repository with the dataset identifier IPX0003347000/PXD032661.

Field-specific reporting

Please select the one below that is the best fit for your research. If you are not sure, read the appropriate sections before making your selection.

☒ Life sciences ☐ Behavioural & social sciences ☐ Ecological, evolutionary & environmental sciences

For a reference copy of the document with all sections, see nature.com/documents/nr-reporting-summary-flat.pdf

Life sciences study design

All studies must disclose on these points even when the disclosure is negative.

Sample size	No sample-size calculation was performed.
Data exclusions	No data were excluded from the analyses.
Replication	For image, biochemistry, and cellular experiments, all attempts at replication were successful. For mass spectrometry experiments, the replications were analyzed in paper.
Randomization	N/A
Blinding	N/A

Reporting for specific materials, systems and methods

We require information from authors about some types of materials, experimental systems and methods used in many studies. Here, indicate whether each material, system or method listed is relevant to your study. If you are not sure if a list item applies to your research, read the appropriate section before selecting a response.

Materials & experimental systems

n/a	Involved in the study	
☐	☒	Antibodies
☐	☒	Eukaryotic cell lines
☒	☐	Palaeontology and archaeology
☒	☐	Animals and other organisms
☒	☐	Human research participants
☒	☐	Clinical data
☒	☐	Dual use research of concern

Methods

n/a	Involved in the study	
☒	☐	ChIP-seq
☐	☒	Flow cytometry
☒	☐	MRI-based neuroimaging

Antibodies

Antibodies used	Detecting reagents used for western blotting include: streptavidin-HRP (Cell Signaling, 3999S), anti-Myc (Cell Signaling, 2276S), anti-V5 (Abcam, ab27671), anti-β-actin (GenScript, A00702), anti-FLAG (GNI, GNI14110-FG), anti-PMP70 (Sigma, P0497), anti-MFN1 (Cell Signaling, D6E2S), anti-MFN2 (Cell Signaling, D1E9). Antibodies used for immunofluorescence include: anti-MFN1 (Cell Signaling, D6E2S), anti-MFN2 (Cell Signaling, D1E9), anti-calnexin (Abcam, ab22595), anti-EEA1 (Cell Signaling, C45B10), anti-GM130 (Abcam, ab52649), anti-LAMP1 (Cell Signaling, D2D11), anti-PMP70 (Sigma, SAB4200181), anti-PMP70 (Sigma, P0497), anti-catalase (Merck/ Millipore, 219010), anti-FLAG (GNI, GNI14110-FG), anti-FLAG (Proteintech, 20543-1-AP), anti-Myc (Cell Signaling, 2276S), anti-COX4 (Proteintech, 11242-1-AP).
Validation	All antibodies used in this study are commercially available antibodies and have been used extensively in previous research.

Eukaryotic cell lines

Policy information about cell lines

Cell line source(s)	HeLa cell line (ATCC, CCL-2)<br>HEK-293T cell line (ATCC, CRL-1573)
Authentication	None of the cell lines used were authenticated.
Mycoplasma contamination	All cell lines tested negative for mycoplasma contamination.
Commonly misidentified lines (See ICLAC register)	No commonly misidentified cell lines were used.

Flow Cytometry

Plots

Confirm that:

- [ ] The axis labels state the marker and fluorochrome used (e.g. CD4-FITC).
- [ ] The axis scales are clearly visible. Include numbers along axes only for bottom left plot of group (a 'group' is an analysis of identical markers).
- [ ] All plots are contour plots with outliers or pseudocolor plots.
- [x] A numerical value for number of cells or percentage (with statistics) is provided.

Methodology

Sample preparation	Cultured cells were harvested, then washed and suspended in PBS for flow cytometry sorting.
Instrument	BD Fortessa
Software	BD FACSDiva was used to collect data
Cell population abundance	Cells used in this study are all cell lines transected with fluorescent proteins(PerMit Venus).
Gating strategy	In general, cells are first gated with SSC/FSC to select single cells, then gated with YFP channel for positive PerMit Venus HeLa cells. This population is then analyzed for PerMit Venus signal.

- [ ] Tick this box to confirm that a figure exemplifying the gating strategy is provided in the Supplementary Information.
